# Idiopathic combined retinal vessels occlusion in a pregnant woman: a case report

**DOI:** 10.1186/s13256-022-03421-8

**Published:** 2022-05-15

**Authors:** Mohammadreza Mehrabi Bahar, Seyedeh Noushin Ghalandarpoor-Attar, Azadeh Shabani, Sedigheh Hantoushzadeh, Seyed Ali Tabatabaei, Seyedeh Mojgan Ghalandarpoor-Attar

**Affiliations:** 1 Farabi Eye Hospital, Retinal Services, Tehran University of Medical Sciences, Tehran, Iran; 2 Obstetrics and Gynecology Department, Baqyiatallah Hospital, Baqyiatallah University of Medical Sciences, Tehran, Iran; 3 Obstetrics and Gynecology Department, Taleghani Hospital, Shahid Beheshti University of Medical Sciences, Tehran, Iran; 4 Obstetrics and Gynecology Department, Imam Khomeini Hospital Complex, Vali-Asr Hospital, Tehran University of Medical Sciences, Tehran, Iran; 5 Obstetrics and Gynecology Department, Baharloo Hospital, Tehran University of Medical Sciences, Tehran, Iran

**Keywords:** Ocular vascular occlusion, Central retinal vein occlusion, Pregnancy, Thrombophilia, Optical coherence tomography, Angiography

## Abstract

**Background:**

Ocular vascular occlusion is an extremely rare event, especially in the young population. This diagnosis is always associated with active systemic diseases in young adults and needs thrombophilia workup. Nevertheless, we present the case of a pregnant woman suffering from idiopathic combined central retinal vein and cilioretinal artery occlusion.

**Case presentation:**

A 36-year-old pregnant Iranian woman at the 36th week of her second pregnancy complained of subacute unilateral painless decreased vision of her left eye. She had experienced a transient vision loss that lasted several minutes, but attacks gradually became more frequent and finally persistent over a several-day period. Finally combined central retinal vein and cilioretinal artery occlusion was established. Her visual acuity improved to 4/10 in a month without any ocular intervention except for a short duration of prophylactic dose enoxaparin, and the acuity reached 8/10 without any complications in the third month follow-up visit. At 1 year follow-up, the visual acuity had not changed and no macular edema was seen.

**Conclusions:**

Ocular vascular occlusion is extremely rare among young adults, and even rarer among pregnant women. According to this, any suspected retinal vascular event in this population should raise suspicion for underlying diseases such as hypertension, diabetes, autoimmune diseases, migraine, preeclampsia syndrome, and thrombophilia. However, as seen in this presented case, idiopathic ocular vascular occlusion events can occur also.

## Background

Pregnancy by itself is an etiological cause for multiple physiologic and nonphysiologic changes in the women’s body. Among these significant alterations are changes in the hemostatic system, which can predispose women to thromboembolic events such as deep vein thrombosis (DVT), pulmonary embolism (PTE), and much less commonly thrombosis in ocular circulation [1]. However, ocular vascular occlusion (OVO) is extremely rare even in the nonpregnant population, and usually associated with active systemic disorders such as hypertension, diabetes, thrombophilia, autoimmune diseases, migraine, etc. [2, 3].

Although there is an increased risk of thromboembolism during pregnancy, especially in the third trimester and postpartum period, many such affected women are carriers of hereditary or acquired thrombophilia [4]. So, an extensive hematological investigation regarding these predisposing conditions is essential.

As mentioned above, thrombosis risk increases 2–4 folds during pregnancy [5]. Besides this, some other physiological changes occur in the eyes during pregnancy, such as increased lens curvature, decreased conjunctival capillaries, eye dryness, decreased corneal sensitivity, and intraocular pressure. Some changes will also occur in a pregnant woman’s visual field (bitemporal concentric visual field defects) [6]. It is worth saying that, surprisingly and despite these effects, generally, an uncomplicated pregnancy is rarely associated with any significant induced changes in the retina, and less so in retinal vessels. Most pregnancy-related retinal changes are associated with the preeclampsia–eclampsia spectrum [7]. In fact, retinal vessel changes are found more frequently on funduscopic examination of this population [8], but only approximately one-third will experience clinically significant ocular sequelae [9].

There are just a handful of cases of pregnant women diagnosed with OVO in literature.

Ozdamar *et al.* reported a case of right eye cilioretinal artery occlusion (CLRAO) and incomplete central retinal vein occlusion (CRVO) in a patient just a week after elective cesarean section for twin gestation. Fortunately, her ocular and laboratory abnormalities rapidly disappeared 1 month after delivery under conservative treatment [10]. Humayun *et al.* also reported a 32-year-old woman at 37 weeks of gestation with both papillophlebitis and branch retinal artery occlusion diagnosis simultaneously [11]. Papillophlebitis is supposed to be a type of central retinal vein occlusion among young people, while its exact etiology is yet to be well established [12]. There are several reports of CLRAO and CRVO in nonpregnant patients [13, 14], although such reports in pregnancy are extremely scarce.

According to the rarity of the disease in young adults, especially in the pregnant subpopulation, early diagnosis and appropriate subsequent interventions are very important during pregnancy. Considering the few anecdotal case reports [15–17] and short case series [18], knowledge is limited in this era. We present herein the case of a pregnant woman at the 36th week of gestation with diagnosis of combined CRVO and CLRAO. We describe what happened to her during this event and also share our acquired experience with other interested physicians to add more evidence on this topic to the literature.

## Case presentation

**Fig. 1:**
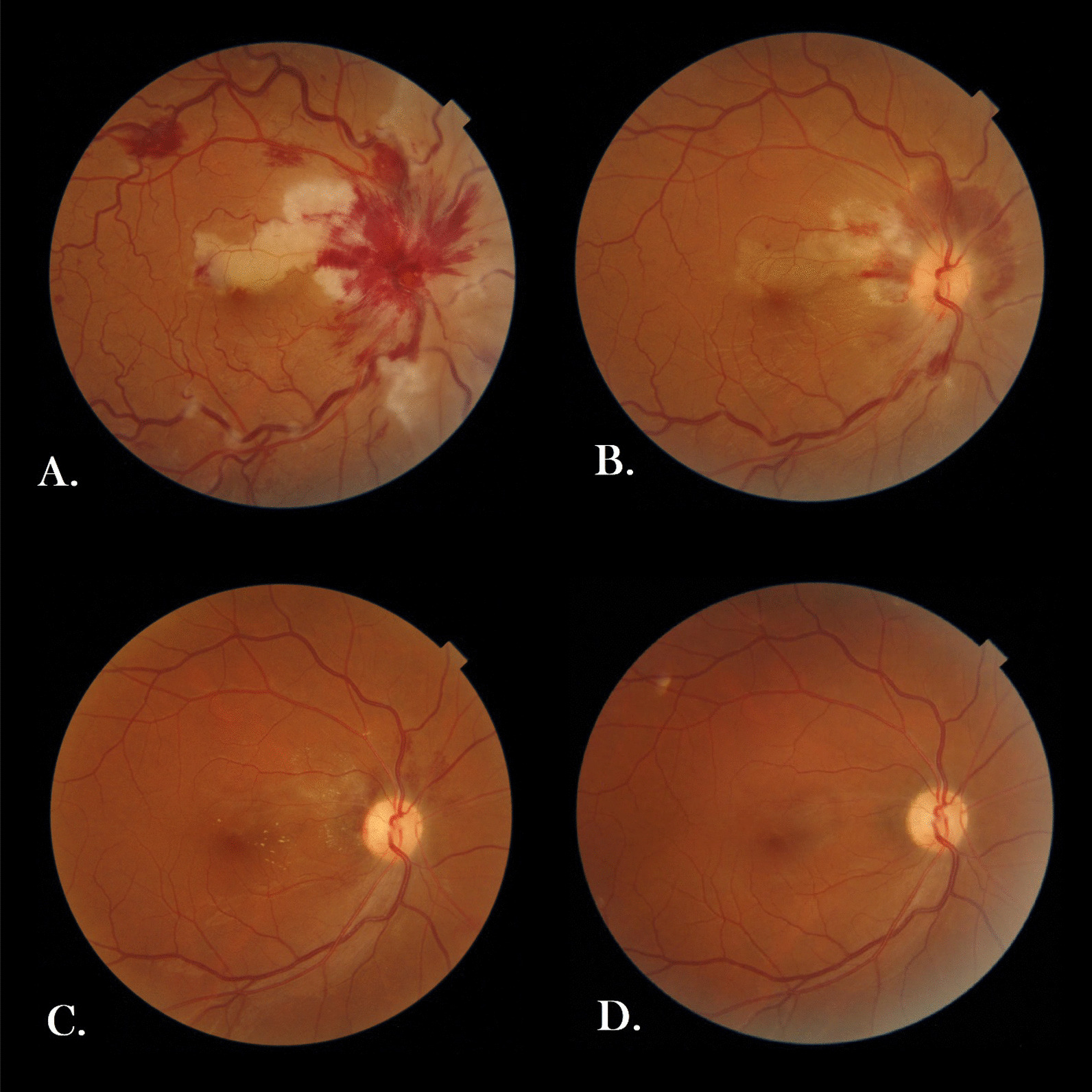
Fundus photo of the patient. **A** Fundus photo of the right eye revealed optic nerve edema and diffuse peripapillary flame-shape hemorrhage and cotton-wool spots in the peripapillary region as well as tortuous and dilated veins, and whitening in superior of the macula in favor of cilioretinal artery occlusion. **B** 2-Week postpartum follow-up and **C** 8-week postpartum follow-up revealed a decrease in hemorrhage and cotton-wool spots and discrete exudate in the macula. **D** At 1-year follow-up, all hemorrhage and exudate had resolved and cilioretinal artery occlusion was barely visible visible at the superior macula

**Fig. 2:**
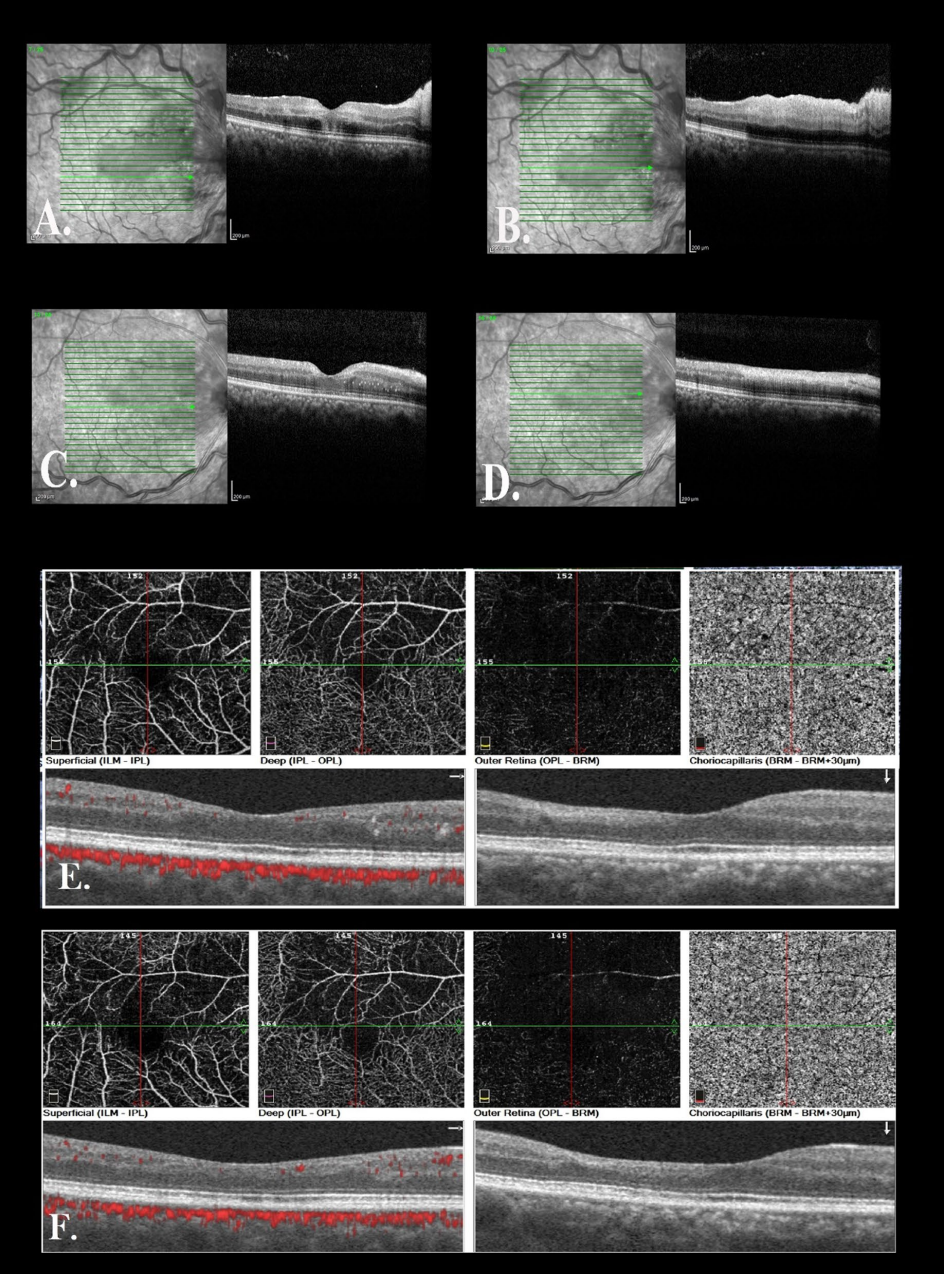
**A** Spectral-domain optical coherence tomography (SD-OCT) of the right eye in fovea showed hyperreflectivity in the middle retinal layer and no apparent edema, and **B** in superior macula revealed thickening and hyperreflectivity of the inner retina and shadowing in cilioretinal artery occlusion area. **C** and **D** At 2-week postpartum follow-up. **E** OCT angiography (OCTA) of the right eye at 8-week postpartum follow-up showed a decrease in vascular density in superficial and deep capillary plexus and no macular edema. **F** OCTA follow-up at 1-year revealed a stable nonperfused area and no macular edema

A 36-year-old gravid2 live1 pregnant Iranian woman at the 36th week of her gestation came to Imam Khomeini (Tehran, Iran) Obstetric Emergency Department in March 2021 complaining of subacute visual disturbance. She had well-controlled gestational diabetes (on diet) in her current pregnancy and history of hypothyroidism since 10 years ago, soon after her first delivery, which was well controlled before and during pregnancy by taking 150 microgram oral levothyroxine daily. She also denied any cigarette smoking or alcohol consumption, and she did not take any other medication except for daily levothyroxine, multi-prenatal, and ferrous sulfate tablets (each one tablet per day). She was a housewife living in a small county in Tehran Province with middle socioeconomical status. Regarding her family history, no thromboembolic event or similar visual or neurological disorders had been evident in her first- or second-degree relatives. She had been suffering from decreased vision that she described as central scotoma since 5 days ago. Her first visual attack had lasted for nearly 15 minutes, but thereafter, similar attacks had reoccurred frequently, finally leading to persistent decreased vision since 2 days before her arrival. She had been hospitalized in another center for 2 days. Brain magnetic resonance imaging (MRI) and neurologic consult were done there, and the first impression was papillophelebitis at that center, but the patient had discharged home at her own request and referred to our hospital for further assessment. Upon her admission, she was completely conscious, her vital signs were all within normal ranges (blood pressure: 115/75 mmHg, pulse rate: 88 beats/minute, respiratory rate: 14/minute, oral temperature: 36.9 °C), and she had no complaints of poor signs (headache, nausea, vomiting, and/or epigastric pain) other than visual disturbance. Her body mass index was 24.9 kg/m^2^, and obstetrical examination was unremarkable (no evidence of uterine contraction or hypertonicity, rupture of membranes, vaginal bleeding, or any abnormalities in fetal heart rate tracing). Moreover, reviewing different body systems, sensorimotor neurological examination, gate assessment, lung and heart auscultation, musculoskeletal and dermatological examination, and also liver and spleen palpation (as far as doable due to presence of a large gravid uterus), no pathological findings were revealed. On ophthalmological examination, uncorrected visual acuity was counting fingers at 3-meter distance and 10/10 in her right and left eye, respectively. Direct fundoscopy of the right eye revealed evidence of retinal ischemia and scattered hemorrhages as well as optic disc swelling. Previous brain MRI was reviewed by another radiologist, and no pathologic findings were documented. Furthermore, no abnormalities were found on carotid sonographic examination or transthoracic echocardiography (TTE). Subsequently, we consulted with an ophthalmologist in Farabi Eye Hospital. Intraocular pressure (IOP) was 13 mmHg in both eyes, and there was no abnormal lesion in anterior segment, as well as no vitritis in both eyes. Posterior segment assessment in her right eye revealed evidence of retinal ischemia, cotton-wool spots, and scattered flame-shaped hemorrhages, as well as optic disc swelling, in favor of CRVO and CLRAO (Fig1A). The next day, spectral-domain ocular coherence tomography (SD-OCT) confirmed our clinically suspected diagnosis (Fig 2A, B). According to the ophthalmologist's opinion, as no macular edema was evident, no further interventions or early delivery was necessitated. The ophthalmologist highly recommended us to check thrombophilia disorders or other relevant systemic diseases as potential predisposing factors and also to rule out preeclampsia syndrome. Additionally, they emphasized continuing prophylactic dose of low-molecular-weight heparin (LMWH) 40 mg subcutaneously daily, which had been initiated on her admission, and they arranged an outpatient follow-up a week later. As our investigations ruled out potential comorbidities and preeclampsia syndrome (all blood pressure measurements were normal, and there was no abnormality in laboratory tests except for D-dimer and CRP) (Table 1), she was discharged home and no drugs except for LMWH were prescribed for an additional 7 days. There were no changes in her symptoms, and visual acuity remained stable at 3-meter counting fingers at 1-week follow-up. Six days later, she experienced labor pain and thus was admitted again at Imam Khomeyni Hospital; due to establishment of active labor, she subsequently underwent an uncomplicated emergency cesarean section through a Pfannesteil skin incision under regional anesthesia, and a 3300-g female newborn with Apgar score of 9 and 10 at the first and fifth minute was delivered. The baby’s height and head circumference were also 51 and 35.5 cm, respectively. Finally, both mother and baby were discharged home 48 hours later without any complications. We strongly recommended her to organize an ophthalmologist’s appointment as soon as possible. Finally, she visited her ophthalmologist 2 weeks after delivery; the follow-up examination and imaging results are shown in Figs. 1B and 2C, D. She had improvement in visual acuity to 4/10 and somewhat decrease in retinal findings. At 8-week postpartum follow-up, her visual acuity was 8/10 in the right eye (Fig. 1C) and ocular coherence tomography angiography (OCT-A) was performed, revealing no edema as well as a decrease in vascular density in cilioretinal artery supply in superior of the macula (Fig. 2E). She had no complications such as IOP rise or neovascularization. One-year follow-up examination was done by the same ophthalmologist, showing that all hemorrhage and exudate had resolved and cilioretinal artery occlusion was barely visible at the superior macula, while on imaging, no macular edema was seen (Figs. 1D, 2F). Moreover, thyroid stimulating hormone, complete blood cell count, and 75-g glucose tolerance test were all within normal limits.

**Table 1:** Patient’s laboratory tests

Lab	Result	Lab	Result
WBC	8400/mL (75% neutrophils)	AST	33 IU/L
Hb	10.9 g/dL	ALT	31 IU/L
Plt	212 × 10^3^/mL	LDH	400 IU/L
D-Dimer	3.5 mg/L (high)	TSH	0.6 mIU/mL
ESR	40 mm/H (high)	ANA	Neg
Anti-dsDNA	8.6 (Neg)	APS pannel	Neg
Plasma homocysteine level	4.75 (Nl)	24-hour urinary protein excretion	175
Protein C level	81 (Nl)	Protein S level	53 (minimum of 42 is normal in the laboratory reference’s range)
Prothrombin gene mutation	Wilde type	Factor Leiden mutation	Wild type

*WBC* white blood cell, *Hb* hemoglobin, *Plt* platelet, *ESR* erythrocyte sedimentation rate, *anti-dsDNA* anti-double strand deoxyribonucleic acid, *AST* aspartate aminotransferase, *ALT* alanine aminotransferase, *LDH* lactate dehydrogenase, *TSH* thyroid stimulating hormone, *ANA* antinuclear antibody, *APS* antiphospholipid syndrome

## Discussion

We describe a rare case of combined CRVO and CLRAO during late third trimester of an uneventful singleton pregnancy. Finally, no underling systemic disorders, specifically thrombophilia, gestational hypertension, or preeclampsia syndrome, were confirmed, which is extremely rare and unreported in literature. Her ophthalmic workup revealed no macular edema, and her vision improved significantly in postpartum period without any systemic or local ocular medications such as corticosteroids. Also, a 1-year follow-up was arranged, revealing no neovascularization or macular edema. Such long-term follow-up seems to be lacking from previous reports.

In fact, normal pregnancy may displace the hemostatic balance toward hypercoagulability. In other words, pregnancy and the subsequent puerperium represent the classical Virchow’s triad components: hypercoagulability, venous stasis, and vascular damage. The hypercoagulable states within pregnancy could be caused by increased thrombin levels and procoagulant factors and decreased endogenous anticoagulant protein S. On the other hand, venous stasis is caused by progesterone effects on blood vessel walls, even in normal pregnancy [19, 20]. Many studies have reported an increased risk of thrombotic disorders during pregnancy [1]. Thromboembolic events rarely occur in the eyes of a pregnant woman unless the pregnancy is complicated, most frequently by pregnancy hypertension [21]. Retinal vein occlusion (RVO), a sight-threatening thrombotic condition, might also be among these thrombotic conditions. Despite the few case reports of RVO in pregnancy, some authors consider pregnancy as a risk factor for RVO [22-24]. Nevertheless, an interesting population-based study on Korean people showed that preeclampsia/eclampsia is a risk factor for RVO during pregnancy, and the authors suggested that pregnancy itself may not be a predisposing condition for RVO [25]. However, ocular vascular occlusive (OVO) events, including retinal vein occlusion (RVO) or retinal artery occlusion (RAO) or combined RVO and RAO, are strongly associated with thrombophilia in young patients [26-28], leading to severe visual problems as seen in our patient, but fortunately, our patient regained much of her vision within 3 months, and her extensive hematologic workup was unremarkable again.

Kurtz *et al.* described three cases of OVO during pregnancy or the immediate postpartum period in which inherited thrombophilia was finally confirmed, in two pregnant women by diagnosis of BRAO at the 8th and 13th week of gestation and in another with history of uninvestigated visual disturbance 4 days after her last delivery with subsequent CRVO event 15 years later. In two patients, protein S deficiency was finally confirmed [29]. It is known that free protein S decreases rapidly in the second trimester of pregnancy, but its level is comparable to nonpregnant females in the first trimester, which is why protein S was in lower normal limit in our case.

However, in contrast to the above-mentioned studies, we could not find any inherited thrombophilia, and there was no evidence (for example, hypertension, proteinuria, and disturbed hepatic or renal function tests) in favor of preeclampsia in the presented case.

Our patient showed combined vascular occlusion (CRVO + CLRAO), and she was younger than the mean age that Reval *et al.* reported in a series of CVOs, although they reported that 10 out of 17 patients developed neovascular glaucoma at 3-month follow-up. Fortunately, there was no neovascular glaucoma evident in her follow-up visits around 3 months and 1 year after her visual problem onset. She also subjectively confessed that her visual disturbance and scotoma had decreased to some extent. At her 3-month follow-up visit, her visual acuity increased to 8/10 Snellen acuity, and she underwent OCT and OCT-A, which revealed no signs of macular edema. On fundoscopic examination, no neovascularization was evident, previous retinal hemorrhage had resolved completely, and retinal and optic nerve head edema had regressed significantly, too. Furthermore, no neovascularization was found 9 months later on her last examination.

Humayun *et al.* also reported a 32-year-old woman at 37th week of gestation who suddenly suffered blurred vision and paracentral scotoma in her left eye. They reported she had papillophlebitis combined with branch retinal artery occlusion. The patient underwent elective cesarean section 3 days after, then received a 3-day course of intravenous methylprednisolone followed by oral prednisolone for 6 months. Her visual field further improved with only a few residual central and paracentral defects. Subsequently, administration of oral prednisone was tapered slowly and finally discontinued 2 months later [11]. In contrast to Humayun’s patient, our patient was just on conservative treatment, except for a short course of prophylactic LMWH (40 mg daily) during admission and a week later. There was some recovery in her first postpartum examination, and macular edema had disappeared too. In fact, in case of CRVO, many treatment options have been introduced, and high-quality studies have shown clinically significant improvement in its sequelae, especially macular edema. In the case of macular edema secondary to RVO, the first therapeutic choice is anti-vascular endothelial growth factor (anti-VEGF) administration [30, 31]. Nevertheless, there is concern about potential risks of this drug during pregnancy. Although no specific guidelines for treating macular edema in pregnant women exist, corticosteroids might be used [32]. However, as the main indication for treatment in such cases of combined CRVO and CLRO is macular edema [30, 33] and our patient did not show any significant macular edema and was also in late pregnancy, no anti-VEGF or corticosteroids were prescribed.

It is worth noting the interesting similar report of the case of a 25-year-old gravid female diagnosed with right eye CLRAO and incomplete CRVO a week after elective cesarean for twin gestation, in whom symptoms disappeared completely 1 month later without any treatment [10]. However, in contrast to our case, that patient had minimal vein occlusion signs, for example, dilated and tortuous veins and few retinal hemorrhages, and despite this, she had 20/20 VA. Interestingly both their and our case had elevated D-dimer serum levels. In fact, D-dimer is the most specific marker of fibrinolysis, which is elevated in presence of thrombosis. On the other hand, an increase in maternal plasma D-dimer level may be seen in normal or complicated pregnancies as well as in the postpartum period. Hence, an elevated D-dimer level in pregnancy does not necessarily indicate a complication [1, 2, and 34].

The last interesting point to consider regarding this case is that our patient experienced an initial sudden attack of central visual disturbance for approximately 15 minutes, subsequently followed by more frequent but shorter-lasting attacks, and finally persistent decreased vision. One hypothesis could be suggested concerning the pathogenesis of decreased perfusion of CLRA, which originates from the choroidal artery, which leads to lower pressure in contrast to retinal arterial supply, and even though CLARO occurred due to more occlusion of the central retinal vein in our case.

## Conclusion

We report the case of a young pregnant woman in her late third trimester who developed combined CRVO and CLRAO. To the best of our knowledge, this report is an extremely rare case of OVO in pregnancy in which we could not find any underlying etiology except for the hypercoagulable state of pregnancy. As well, a 1-year follow-up was performed, revealing no macular edema. Finally, we should state that, although OVO events are very rare during pregnancy, any clinicians involved in pregnant women’s health should take specific consideration regarding this diagnosis in any patient complaining of visual-related signs and symptoms.

## Data Availability

All data generated or analyzed during this study are included in this published article (and its additional information files).

## Abbreviations

OVO: Ocular vascular occlusion
CRVO: Central retinal vein occlusion
CLRAO: Cilioretinal artery occlusion
DVT: Deep vein thrombosis
PTE: Pulmonary thromboembolism
MRI: Magnetic resonance imaging
TTE: Transthoracic echocardiography
IOP: Intraocular pressure
SD-OCT: Spectral-domain ocular coherence tomography
LMWH: Low-molecular-weight heparin
OCT-A: Ocular coherence tomography angiography
RVO: Retinal vein occlusion
RAO: Retinal artery occlusion
Anti-VEGF: Anti-vascular endothelial growth factor
